# Differentiating the role of alcohol in young men’s use of substances with sex: A qualitative study

**DOI:** 10.1186/s12954-023-00835-5

**Published:** 2023-07-31

**Authors:** Trevor Goodyear, John L. Oliffe, Natasha Parent, Caroline Mniszak, Emily Jenkins, Rod Knight

**Affiliations:** 1 British Columbia Centre on Substance Use, 400-1045 Howe St, Vancouver, BC V6Z 2A9 Canada; 2 School of Nursing, University of British Columbia, Vancouver, Canada; 3 Department of Nursing, University of Melbourne, Melbourne, VIC Australia; 4 Department of Medicine, University of British Columbia, Vancouver, Canada; 5 École de Santé Publique, Université de Montréal, Montréal, Canada

**Keywords:** Men, Youth, Sexual minorities, Alcohol, Sex, Sexualized substance use, Sexual health, Harm reduction

## Abstract

**Background:**

Alcohol consumption is common among young men and occurs in many contexts. The sexualized substance use literature has generated some insight into the role of alcohol alongside other substances in the context of sex, though there remain opportunities for targeted and context-sensitive studies to examine the sexual practices and outcomes associated with alcohol, specifically.

**Methods:**

This interpretive description study explores how experiences and contexts of alcohol use feature in the sexual lives of young men who use substances with sex. Data collection involved in-depth interviews conducted between 2018 and 2021 in Vancouver, Canada, with *N* = 76 young men (ages 18–30; mean = 23.9 years) who use substances with sex, including men with diverse sexual identities. Data were analyzed in an iterative manner through a social constructivist lens and an interpretive description framework, leveraging constant comparison techniques.

**Findings:**

This analysis yielded three interconnected themes: (1) using alcohol for sexual(ity) freedoms; (2) backgrounding alcohol within a sexualized polysubstance milieu; and (3) navigating the risks and consequences of using alcohol with sex. Alcohol use was found to reduce inhibitions and support experimentation, including by facilitating the transgression of conservative or restrictive social and sexual norms. Alcohol was seldom explicitly classified as a sexualized substance, though it was evidently a widespread and normative social practice. This practice was associated with important risk and consequences, including with respect to consent, pregnancy and sexually transmitted and bloodborne infection risk, and sexual dysfunction.

**Conclusions:**

Findings from this study position alcohol as a backgrounded yet significantly influential substance that young men use with sex. The sexualized use of substances must be understood, and responded to, in a manner that is grounded in harm reduction and that considers the full spectrum of substances—inclusive of but not limited to alcohol—and associated benefits and risks that feature in young men’s sexual lives. Specifically, sexual health and primary care providers working with young men should invite and open up meaningful conversation about how they may be using substances (including alcohol) with sex, while offering de-stigmatizing, sex-positive, and affirming education and supports to promote safer sex and substance use.

## Introduction

Research at the intersections of substance use and sexual health represents a growing field of inquiry, with scholars working to understand strategies to reduce harms that can arise among men who use substances with sex [1]. For example, epidemiological studies have examined the interconnections between substance use practices, sexual behaviors, and sexually transmitted and bloodborne infection (STBBI) risk among sexual minority men—that is, cisgender and transgender gay, bisexual, queer, and other men who have sex with men [1–5]. Research has also generated understandings of the benefits and risks associated with sexualized substance use among sexual minority men, as well as strategies for risk-mitigation [4]. Within this body of work, some configurations of sexualized substances are relatively well studied, notably in research on chemsex or Party n’ Play (PnP)—that is, the (co-)use of stimulants (e.g., crystal methamphetamine, MDMA, cocaine), alkyl nitrites (poppers), ketamine, and/or GHB to enhance sexual experiences [1, 4]. Still, there are opportunities for further research, including with studies that seek to delineate the roles of specific substances in these contexts whilst developing responsive policy and practice recommendations. The current study seeks to extend knowledge in this area by exploring young men’s [18–30 years] experiences and contexts of using alcohol with sex.

## Background

Alcohol use is a priority men’s health issue. Rates of alcohol use have historically been high among men [6], with subgroups of men experiencing inequities in patterns of alcohol use and associated risks, including men who are young, trans, and sexual minorities [7–9]. In Canada, where the current study is set, for example, young men between the ages of 18–34 experience the highest rates of both drinking and “heavy drinking”—that is, having four or more (for females) or five or more (for males) drinks on one occasion, at least once a month during the past year [10]. Data such as these suggest the importance of alcohol-related research that focuses on men, and that examines population-specific mechanisms and contexts of use.

Population-level data about men’s alcohol consumption are themselves concerning, but so too are potential harms associated with alcohol use and the contexts in which it occurs. This is especially so with respect to young men, for whom scholars have theorized intersections between alcohol consumption practices and masculine norms implicated in risk-taking, including with sex [1, 4, 5, 11, 12]. Together with literature on the complex relationship between alcohol use and sexual health risks, qualitative studies and knowledge syntheses have provided some insight into young men’s motivations, experiences, and contexts of sexualized alcohol use. For example, the perceived functional benefits of using alcohol with sex have been reported, such as with young men using alcohol to feel more confident and to enhance sexual satisfaction and pleasure, while more generally facilitating socialization and enjoyment [13, 14]. These findings are supported by knowledge syntheses about sexualized substance use among young men with minoritized sexual identities [1, 4, 15], which thoroughly characterize how young men (and other groups) perceive and experience sexualized substance use across multiple domains (e.g., physical, mental, emotional, social).

Despite their important contributions, existing knowledge syntheses about young men’s sexualized substance use offer less in the way of *alcohol-specific* insights. This is a noteworthy research gap, not only because different substances and/or configurations of substances have distinctive effects, but also because of the diverse ways and contexts in which substances are used. With sexualized alcohol use, there remain notable literature gaps surrounding how diverse young men consider and navigate alcohol-specific harms and harm reduction. At this juncture, targeted and context-sensitive studies are needed to generate in-depth understandings about how alcohol features in young men’s sexual lives, whilst informing interventions and care efforts to mitigate potential harms and promote sexual health and well-being. As such, this study contributes to the literature a focused exploration of *how*, *why*, and *with what effects* young men are consuming alcohol with sex, while examining these phenomena in relation to young men’s broader experiences with sexualized substance use. Specifically, the objective of this study is to explore how experiences and contexts of alcohol use feature in the sexual lives of young men who use substances with sex.

## Methods

### Study approach

This qualitative study follows an interpretive description research design [16]. Interpretive description is a pragmatic and inductive methodology for investigating health-related issues and generating practice-oriented findings [16]. Consistent with interpretive description, we situate this study within a social constructivist paradigm [17] that aims for knowledge co-production, including by privileging and staying true to research participants’ descriptions of their lived experiences whilst also leveraging researchers’ social (re)constructions and syntheses of these accounts. With this study approach, we sought to describe and contextualize young men’s use of alcohol with sex while exploring the social meaning they assign to sexualized alcohol use, the social processes through which these experiences take shape, and the socio-political and subjective contexts in which we (as researchers) interpret these experiences.

### Sampling and recruitment

This study is nested in a larger program of research examining young men’s use of substances (inclusive of but not limited to alcohol) with sex. Set in Vancouver, Canada, we used a stratified purposive approach [18] to recruit a sexually diverse sample of young men who use substances with sex, stratified by sexual orientation (sexual minority men; heterosexual men). Study advertisements were posted on social media and in a variety of in-person clinical and community locations. Prospective participants were eligible for inclusion if they lived in Vancouver, spoke English, self-identified as men, were 15–30 years old, had previously been or were currently sexually active, and had used substances with sex. Participants provided informed consent and received a CDN $30 honorarium. Approval was granted by the University of British Columbia Behavioural Research Ethics Board (#H16-01915-A013).

### Data collection

Between January 2018 and February 2021, we conducted 76 in-depth, semi-structured interviews lasting 1–2 h each. Most interviews were held in person at our offices in downtown Vancouver; ten were conducted remotely over Zoom, following the onset of the COVID-19 pandemic. Using a semi-structured guide, interviewers sought to elicit discussion of perspectives and experiences of using substances with sex and how these experiences may intersect with the relational and socio-cultural contexts of young men’s lives. Interviewers also administered a socio-demographic questionnaire while recording participant responses. Interviews were audio-recorded, transcribed verbatim, accuracy checked, and anonymized with participant-selected pseudonyms.

### Data analysis

We used NVivo 12 and Microsoft Excel to manage the data. The early analysis was concurrent with data collection and involved reviewing the initial transcripts to support data familiarization and refinement of a preconfigured codebook, which we had developed by drawing on our past research experiences and our review of the sexualized substance use literature. The present analysis focuses on data coded at both “sexualized use of substances” and “depressants” (including alcohol, toward which we directed our attention). We contextualized this inquiry by pulling and analyzing data from other codes, including “sex, gender, and sexual orientation;” “relationships;” “risk;” “sexual harm reduction practices;” and “pleasure, sexuality, and desire.” Consistent with the logic of interpretive description [16], we leveraged constant comparison techniques to organize data from these conceptual buckets into patterns that we assigned substantive open codes, reflecting high-level generalized categories and analytical insights. These codes were iteratively grouped, collapsed, and reviewed as we deepened our familiarization with the data.

We developed preliminary thematic findings by iteratively redefining, splitting, and combining the initial open codes. Regular meetings between the first and last authors also helped to debrief, construct, and refine key patterns in the data. We consistently returned to the data for nuance and depth, reviewing data pulls and revisiting the full interview transcripts, whilst also theorizing interconnections between the findings and pertinent literature on pleasure, gender/sexuality, harm reduction, and substance use, including in the context of sex [19–21]. This process prioritized our analytical attention toward how these key concepts and ideas featured and intersected within the data. Notably, we did not focus on distinguishing sexual minority men’s experiences from those of cisgender, heterosexual men, as we felt that there was far more congruence *across* these young men’s narratives and because doing so was outside of the scope of our study objective. Indeed, our aim was to construct and refine central themes capturing the prevailing complexities of diverse young men’s experiences and contexts of using alcohol with sex.

## Findings

### Overview

We interviewed a total of 76 young men between the ages of 18 and 30 years (mean = 23.9). Seventy-one participants identified as cisgender men and 5 as transgender men. They identified with a range of sexual orientations, including gay (*n* = 33), bisexual (*n* = 19), straight (*n* = 19), pansexual (*n* = 5), and queer (*n* = 4). Participants identified as white (*n* = 41), Indigenous (*n* = 11), Black (*n* = 7), and other racialized ethnocultural identities (*n* = 27). Participants could select multiple response options when asked about their gender identity, sexual orientation, and ethnocultural identity.

We constructed three interconnected themes from our analysis of the interview data: (1) using alcohol for sexual(ity) freedoms; (2) backgrounding alcohol within a sexualized polysubstance milieu; and (3) navigating the risks and consequences of using alcohol with sex. The first theme explores how young men use alcohol to reduce inhibitions and support experimentation, including by facilitating the transgression of conservative or restrictive social and sexual norms. The second theme complicates these findings by detailing how young men seldom explicitly classified alcohol as a sexualized substance, despite its utilization (i.e., before, during, after) and effects with sex and that alcohol use was a widespread and normative social practice. The third theme further details the role of alcohol with sex by characterizing how young men navigate risks and consequences associated with sexualized alcohol use. These themes are presented below and are supported by illustrative participant quotations.

### “Loosening up”: Using alcohol for sexual(ity) freedoms

During our interviews, participants indicated that consuming alcohol could have powerful effects on reducing their inhibitions and on supporting them to transgress conservative or restrictive social and sexual norms. Participants valued the “social lubricant” properties of alcohol, within—yet also outside of—sexualized contexts. For example, when asked about issues of loneliness and anxiety and how these might intersect with his use of alcohol, one participant shared:

There’s something there. And the whole like drinking to be more social thing, that might be, like, a whole escape to… fitting what is [socially] required, or like what is… viewed as appropriate for being at a party. […] A loss of inhibition probably would be a big one (Tracao, a 23-year-old man who was uncertain about his sexuality but identified as possibly queer, gay, and/or bisexual).

These broader experiences provide helpful context for nuancing understandings of how participants are using alcohol with sex. Indeed, just as young men may consume alcohol to fit in and connect with peers, participants described using alcohol to facilitate comfort and sociability in sexual contexts. Often, participants shared that drinking could help them to “*feel more uninhibited*” in the lead-up to sexual encounters, and to “*get the ball rolling*” in terms of initiating and having sex. In characterizing these benefits, two participants reflected:

I’ve definitely used alcohol, more in the lead-up than anything. […] In general, I tend to be a very socially anxious person, and so, I’ve consumed alcohol to sort of numb that a bit and be a bit more social, because I find when I’m in the right environment it does make me more sociable. So, I’ve never used it for... like the sex specifically, but to make myself more comfortable around other people leading up to, or what may lead up to a sexual encounter (Bryce, a 20-year-old bisexual man).

[I] like to drink for sex. Feeling more free, sort of loosens you up a little bit, you can relax more, have more fun with it. I think specifically for me I don’t see using a substance as, like, a prerequisite for any sexual encounter I might have, but I think [that] when I do drink, I then become more prone to wanting sexual interaction with someone (Connor, a 20-year-old gay man).

Some participants tied the disinhibiting effects of alcohol consumption to feelings of shame and discomfort they associated with their (minoritized) sexual identities. As we detail at length elsewhere while focusing on cannabis use [22], participants occasionally described feeling guilt and shame related to their sexual identities, as well as their non-(hetero)normative sexual relations and practices. Perhaps understandably, these feelings could worsen with and following (queer) sex, yet they could also be mitigated through use of alcohol and substances, even if only temporarily. This was articulated by Avery, a 24-year-old gay man, just after he finished characterizing the internalized homophobia, guilt, and shame he carried while growing up in a socially conservative community:

I carry a lot of guilt and shame about that. That is quite unfortunate. And so, when you want to be enjoying something [participant referring to sex] with somebody, you want to just enjoy it and be a normal person. And so, it’s walking that line. […] [After sex], as well. Like, that’s when I would feel ashamed normally. But if you’re under some sort of substance then you, you know, even afterwards you feel okay. […] By the morning, [participant trips on his words] I’ll be sober and not… you know, usually there could be that window of shame afterwards.

Participants frequently coupled the disinhibiting effects of using alcohol with sex with its transformative potential. They told us how sex under the influence of alcohol could be more adventurous, with lowered inhibitions supporting participants and their sexual partners to “*take things a little bit further,”* including by exploring their sexualities and sexual preferences. This included engaging in sexual practices they might not partake in sober, such as rough(er) sex, fetish play, public sex, experimenting with sex toys, and role-playing and role-reversing during sex. While participants seemed to sometimes (and understandably so) refrain from describing the intimacies of their sexual lives with the interviewers, as we do here in choosing not to report on some of the details that *were* shared, we emphasize that using alcohol supported these young men and their sexual partners to try new things and engage in new forms of play during sex. As Graham, a 22-year-old gay man, put it when speaking to sex under the influence of alcohol and cannabis, “*the boundary of inhibitions are a lot…hazier*” and “*my openness to try ‘sexually taboo’ things is a little bit lowered when I have been intoxicated in some way*.” To this point, another participant shared the following while describing how different types of alcoholic beverages (e.g., beer versus wine) seem to have distinctive effects on his sexual life:

Sometimes if we’re having beer or something, it can weigh us down or whatever, and we’d usually be less inclined to do it [have sex]. But something like wine, or liquor, will tend to make us a little bit more fearless in the bedroom, you know? Like, be inclined to be more experimental and stuff, if we’ve hit that right balance of buzz, you know, not like too drunk to do it. So yeah, it’s really nice if there’s just a bottle of wine or something. And that usually does the trick. [...] I’m more inclined to use [sex] toys and stuff when I’m drinking. […] My partner gets more aggressive [during sex] when they’re drunk, which I really like. I like it when my partner’s aggressive because she’s usually not. […] That just makes it fun for me (Byron, a 22-year-old man who identifies as bisexual and pansexual).

Participants generally approached these experiences of adventure and experimentation with enthusiasm. They spoke with pride about how using alcohol freed them to have the kinds of sex they desired yet otherwise may not have explored, especially among men who were younger or only beginning to experiment with sex. This sort of “*liquid courage*,” as they sometimes framed it, served to enhance young men’s confidence and sense of play, while concomitantly helping them to explore their sexualities and mitigate experiences of sex(uality)-related shame. This was chiefly so for men whose sexualities could be read as counter to conservative or restrictive social and sexual norms, such as those who were having sex with other men and/or who considered their sexual preferences and desires to be “*sexually taboo*.” Using alcohol and other substances facilitated experimentation with sexual(ity) freedoms and with less of the emotional baggage that can accompany certain forms of sex, as summarized by Avery:

[Using substances with sex, people] would do things that they wouldn’t normally otherwise do. And by being inebriated, you would feel fewer of the negative emotions that you might associate with whatever act you’re doing.

### “Not something I go out of my way to do”: Backgrounding alcohol within a sexualized polysubstance use milieu

Overall, participants seldom explicitly classified alcohol as a sexualized substance. In responding to our questions and prompts about alcohol, specifically, they appeared to have trouble situating their use of this substance within broader contexts of (sexualized) substance use. At a fundamental level, some participants did not even identify alcohol as a substance/drug, let alone a “sex drug.” When asked what comes to mind with respect to the word “substances,” they often responded with some iteration of: “*Any drug that isn’t alcohol*.” This seemed attributable, in part, to broader socio-cultural influences on alcohol-related norms. Alcohol use was indeed commonplace and normed within participants’ peer networks and broader communities, which led many to be hesitant to reduce or essentialize it as a *sexualized* substance. This involved an inadvertent discounting of alcohol’s positioning as a sex drug vis-à-vis those which young men perhaps more explicitly and uniquely associate with sex. Young men who used multiple sexualized substances or who do so intensively were especially likely to hold this normative frame; in this study sample, this tended to be young sexual minority men. For example, Tracao (introduced above) offered the following in an interviewer-participant exchange:

**Interviewer:** When you hear the term “sex drugs,” what comes to mind for you?

**Participant:** Poppers and Tina [crystal methamphetamine], particularly.

**Interviewer:** Yeah. Why is that, do you think?

**Participant:** Because those are the ones people use a lot, particularly for sex. Like, liquor I would associate too, but that’s more of a general, as opposed to just a sex drug.

Alcohol did seem to play somewhat of a lesser role when compared to other substances that featured in young men’s sexual lives. Often, participants discussed their use of substances with sex in a broad sense, speaking in large part to the *effects* of sexualized substance use rather than to the substances themselves. They expressed an appreciation for the varied benefits of using different configurations of substances with sex, including to facilitate euphoria, excitement, pleasure, sensation, and disinhibition. These discussions often related to masculine norms around sexual performance and men’s libidos, with participants reporting that they intentionally used substances with sex to facilitate “*stamina*,” “*vigor*,” and “*strength*.” One participant described how doing so can also enhance confidence and more generally make you feel as though “*you can eat the whole world in one bite.*” The benefits that participants associated with sexualized substance use were often described as providing the means to concurrently elevate their sexual prowess and masculine status, as stated by Victor, a 26-year-old bisexual man:

[Using substances with sex] cuts inhibitions short. You know, it cuts your inhibition. […] It makes you feel more in control. It makes you feel more happy to do some stuff that are usually considered weird. It makes you feel like the superman of gay sex!

Participants did not always specify the substances—or configurations of substances—they were using with sex. It was often only through prompting that they would focus in on the nuances of using specific substances with sex. For instance, when talking about his decision-making process for choosing sexualized substances, one man shared: “*If I want to last long [during sex], I use certain drugs; if I want to cum fast—if I want to last short, I use certain drugs, too*.” Whereas other substances (e.g., poppers, GHB, crystal methamphetamine) were often used explicitly for the purposes of transforming and/or optimizing one’s sexual experiences, alcohol use tended to feature in contexts of sexualized polysubstance use in more organic, even happenstance ways. Reflecting on this, one participant summarized that alcohol is “*on the backburner in a lot of ways*,” a sentiment that was echoed across many interviews. Consider for example these contrasting accounts of the planning (or lack thereof) and lead-up to sex with crystal methamphetamine and alcohol, respectively:

The meth is like – I plan it out and it’s like, okay, today I’m gonna get high and, like, be a slut and it’s like, cool! Yeah [laughs] (Gato, a 28-year-old gay man).

[Alcohol], it’s hit and miss. Drunk sex can either be the best sex of your life or just like the sloppiest, most awful mess ever. … I don’t love it; I don’t try to get drunk if I know I’m gonna have sex that night. If it happens, it happens. [. . .] But, you know, it’s not something I go out of my way to do (Ryley, a 19-year-old gay man).

Implicated in normative assessments of alcohol’s status as a “sex drug” were the trade-offs associated with sexualized alcohol use relative to the sexualized use of other substances. Participants tended to emphasize that both the benefits and risks of using alcohol with sex paled in contrast to those associated with other substances. Even when participants explicitly labeled alcohol a “sex drug,” it was clearly positioned as one that plays a less significant role when compared to the potential benefits and drawbacks that they associated with using other substances with sex. Participants underscored that this was because “*the safety concerns [with alcohol] aren’t as high as some of the other substances*,” and because sex with alcohol was perceived to be less pleasurable than sex with other substances. In effect, the young men generally viewed their use of sexualized substances along a spectrum, with alcohol plainly positioned among the least “extreme” substances. This process of categorization was illustrated by Jacob, a 22-year-old gay man, as he described how substances are differentially stigmatized and attributed risk:

I think there’s a sliding scale where it’s, like, alcohol and weed are over here [gesturing to one side with this hands] and then you have, obviously, things like fentanyl is at the very [other] end of the spectrum, and then you have all these things, like heroin, and then you have crack over here, and then you have ecstasy over here [gesturing to the middle].

Some participants—especially those with relatively fewer experiences with chemsex/PnP—differentiated their personal experiences of using alcohol with sex from what they perceived to be the more prominent norm of sexualized substance use in queer/gay communities. These participants resisted labelling their use of substances with sex as “sexualized,” seemingly because of concerns and assumptions they held regarding these (often-stigmatized) phenomena. This was common among participants who were discussing sexualized substance use in the also stigmatized context of queer sex. For example, one bisexual participant separated himself from what he understood to be the dominant culture of chemsex/PnP in queer/gay communities and in sex between men, while nonetheless acknowledging that he uses alcohol when having sex with women. In juxtaposing his experiences of using alcohol during sex with women to his exposure to chemsex/PnP during sex with men, he seemed unable to reconcile the distinct differences in a way that could directly position alcohol as a “sex drug”:

Being someone who also has sex with guys, I’ve seen the gay community and the queer community, like it [using substances with sex] is very prevalent. […] Sex drugs, I don’t… I guess I can’t even see it, in the context of my sexual experiences with women. Like, it just hasn’t been… well, then I guess, again, alcohol and [chuckles] marijuana have definitely been used in that way. But once again, when I think “sex drugs,” I do think about, like, the pill-popping kind of maybe cocaine-style drugs. Personally, I wouldn’t touch that stuff with a ten-foot pole, because it just freaks me out so much (Drake, a 20-year-old bisexual man).

Participants occasionally surfaced reasons for using alcohol with sex that were more deliberate and intentional, yet this tended to be for counteracting the potentially intensive effects of other substances they were using with sex. This includes Salvadore, a 26-year-old bisexual man who described using alcohol strategically to “*keep [himself] level with meth.*” More commonly, however, alcohol use tended to feature in these young men’s sexual lives in more subtle and (from participants’ perspectives) incidental ways. In navigating their day-to-day lives and relational contexts in which consumption of alcohol was commonplace (e.g., casual drinks with friends, going out to bars/clubs, having a glass of wine with romantic/sexual partners), participants posited that it was only natural that there would be occasions wherein they would have sex while intoxicated. For example, similar to Ryley’s comment above that using alcohol with sex is generally “*not something [they] go out of [their] way to do,*” other participants shared:

Alcohol is already at the situation. Like, me and my partner [are] already having drinks - well, me and my ex were already having drinks – and then things get frisky (Jacob, a 22-year-old gay man).

Basically, the way that sex usually happens is, it’s usually, like, us hanging out, a couple glasses of wine, smoking a joint, making out starts happening and then… the fun happens. Yeah. So, it’s very, I don’t know, I won’t say organic, but it’s just very, like… you come home, you relax… yeah. […] Drinking wine doesn’t [always] lead to sex, but definitely more – more times than not (Valentina, a 29-year-old gay man).

It became evident through our analysis that using alcohol in sexual contexts—whether before, during, and/or after sex—was a widespread and normative social practice for this sample of young men. However, it was young sexual minority men specifically who tended to recount leveraging alcohol and polysubstance use for the explicit purposes of maximizing pleasure and sociability in the context chemsex/PnP. This is not to say that cisgender, heterosexual young men do not partake in chemsex/PnP, only that these narratives seldom surfaced in the current study, beyond brief acknowledgement during the socio-demographic questionnaires that they had also used other substances—specifically, cannabis, psilocybin, cocaine, and MDMA—with sex.

### “A very fine line”: Navigating the risks and consequences of using alcohol with sex

From our conversations with participants about sexualized substance use (inclusive of but not limited to alcohol), we were left with the impression that experiences of risk, consequence, and pleasure were viewed as inseparable. As Connor (introduced above) succinctly put it when discussing these mutually dependent issues, “*An increased desire for pleasure will always come a little bit with an increase in risk.*” In general, participants described feeling knowledgeable about how to identify and actively mitigate risks related to (sexualized) substance use. With alcohol specifically, however, participants emphasized that mitigating pertinent risks and consequences could be distinctly challenging. Participants explained that the trade-off between the benefits and challenges of using alcohol with sex was not always worth it, in retrospect:

[There] is the double-edged sword of alcohol, right? Like, it’s more easy to have those [sexual] experiences [when consuming alcohol], but when you get to the time it’s just, you know, less enjoyable. You don’t – your body is less able to, you know, perform or do whatever you want, or it’s just less enjoyable, because you don’t feel things the same way […] And I don’t think it’s worth the risk (Kevin Nguyen, a 27-year-old straight man).

Alcohol will, oftentimes, the end will not justify the means. You’ll have this alcohol that supplemented the situation, but the overall positive aspects are very minimal (Brazil, a 19-year-old man who identifies as straight and pansexual).

Building on this study’s first theme of “loosening up,” Brazil suggests above that the disinhibiting effects of alcohol may indeed facilitate and increase the likelihood of sexual encounters, yet whilst also affecting their quality, sometimes negatively. Read another way, alcohol seemed to feature as a means of fueling young men’s conquests of masculinity, though not without inhibiting ideas for sexual prowess and pleasure in those encounters. Specifically, participants emphasized that sexual encounters under the influence of alcohol could be unsatisfactory when participants were heavily intoxicated, as opposed to being only mildly or moderately inebriated. It was when they bordered on being “too drunk” that the relationship between alcohol-related benefits and risks could become paradoxical in nature, with experiences of pleasure decreasing alongside a rise in foreseeable consequences. Indeed, Brazil (above) went on to explain how sexualized alcohol use’s threshold for going from good or neutral to bad is a “*very fine line and it’s very variable*,” though, when drinking, “*your brain will think of it as a very wide range*.” Another participant summarized, “*If you consume a lot of alcohol, to the point where you get drunk, it’s very hard to think straight and make rational decisions*.” Elaborating on this perspective, Avo Cado, a 20-year-old gay man, shared the following when asked about how substance use may influence decision-making around safer sex practices:

With things like alcohol, yeah, you make terrible decisions and you’re not, like, completely conscious. […] The more drunk you get, the more you forget. Or, like, forget to control yourself and then you keep drinking, and it’s so easy just to take a couple of shots and then all of a sudden you’re black out [drunk] or something. I feel like alcohol is the evil one out of everything [relative to other drugs]. […] Being aware, and choice-making, I think alcohol is worse, definitely.

At times, participants acknowledged the potential that “*people might end up doing something that they regret”* when having sex while intoxicated and with potentially lowered inhibitions. This was predominantly so regarding their perceived capacities for navigating conversations about, and actions related to, consent in the context of sex under the influence. Included here were concerns about—and in some cases experiences of—having sex that is too rough and that could potentially cause unwanted bleeding, being coerced and taken advantage of, and explicitly being the victim and/or perpetrator of sexual assault. Dynamics between experiences of disinhibition and consent when using substances (including alcohol) with sex were indeed raised by participants, a point to which we will return in our discussion of the study findings.

On the topic of sexual health in the context of sex involving alcohol, many participants discussed specific issues and risks with which they were concerned. Here, they indicated that they could be more vulnerable, impulsive, or even laissez-faire with sexual decision-making while drinking. Consistent with risk-taking masculinities, young men’s pursuit and experiences of pleasure could at times take primacy over efforts to mitigate immediate risks. For this reason, some participants described purposefully avoiding or closely monitoring (and reducing) their use of alcohol when they suspected they may later be having sex. Still, for some, the often-unplanned nature of sex under the influence of alcohol made it difficult to fully prepare for safer sex. Thus, sexual health-related decisions tended to be made “*in the moment*,” while already intoxicated:

When you’re drunk and you’re thinking to yourself, like, “I should wear a condom,” and then if the girl says like, “Just take it off; it’s not working,” you’re just kind of like, “Yeah, fuck it.” It [the condom] is off. Alcohol is a prime example of that, where you’re not really thinking about the consequences of your actions (Finch, a 24-year-old straight man).

When you’re in the moment, especially when you’re using substances, it can be easier just to say kind of “fuck it” with condoms, or whatever. I guess alcohol would be a big one for that. And yeah, I guess when you’re on substances, you can sometimes do things you wouldn’t normally be comfortable with, or that you wouldn’t normally do (Anthony, a 27-year-old bisexual man).

Important to note is that alcohol—even when consumed heavily—did not always result in sexual health risk practices. Overall, participants seemed highly knowledgeable about and capable of mitigating risks that may arise in the context of sex, including when substances are involved. This was particularly so with sexual minority participants, whose sexual health experiences seemed to be deeply shaped by community histories and individual concerns related to HIV and other sexually transmitted and bloodborne infections. When prompted about his use of substances with sex, one participant emphatically stated, “*No matter how drunk I am, I still use condoms and still, you know, ask—inquire about—their status with STIs and HIV*.” This high level of sexual health literacy and agency was echoed by many other participants and, while admirable, seemed at times to be undergirded by a pervasive phobia about, and stigmatization of, HIV. This was evident in the perspective of Sandra Day O’Connor, a 30-year-old queer man:

The worst thing is HIV. It’s always in the back of your mind if you’re having sex with somebody, whether you’re sober, drunk, or whatever. Like, you’re checking the condom, you’re making sure it’s not ripping or tearing, and it’s always in the back of your mind.

Over and above sexual health risks, participants emphasized that using alcohol with sex had the potential to decrease experiences of pleasure, including by threatening men’s libidos and sexual performances. In general, participants indicated that using alcohol could make them tired and uninterested in sex, with one participant reflecting that being heavily intoxicated with alcohol “*makes you impotent*” because “*it’s a downer*.” More specifically, participants identified that using alcohol with sex could lead to challenges with getting and maintaining an erection, and/or with orgasming. Some examples of the potential sexual drawbacks that can come from using alcohol with sex, including of consuming too much alcohol, were shared by participants:

I definitely have a hesitation towards getting too drunk, for sure. Because that can, you know, inhibit erections and stuff like that. It can make it a lot more difficult, so staying conscious of that (Max Power, a 25-year-old straight man).

You can have problems in terms of getting hard, or cumming, which [exhales] it’s not as common for me to not get hard. I don’t – I usually can get hard, it’s just the cumming part. Like, you just don’t get that same sensation (Brazil, introduced earlier).

In sum, participants described various risks and consequences that they associated with using alcohol in the context of sex, most notably at times when they were intoxicated. These adverse effects can be juxtaposed against the benefits of using alcohol with sex, characterized in the first two themes of this analysis. As Graham, a 22-year-old gay man explained toward the end of his interview, there is a certain “*risk management element of things when we’re talking about sex in the context of substances and risk and pleasure*,” including with alcohol.

## Discussion

This interpretive description study explores the backgrounded yet significant and occasionally transformative ways in which alcohol features in the sexual lives of young men who use substances with sex. Emphasized in this analysis are the “social lubricant” properties of alcohol, with its consumption helping participants to try out new forms of play during sex, while also reducing feelings of anxiety and shame associated with non-(hetero)normative sex. These benefits are juxtaposed against the dynamics of balancing risk and consequences with sex involving alcohol, including with respect to consent, pregnancy and sexually transmitted and bloodborne infection risk, and sexual dysfunction. Together, these findings offer substance-specific insights that hold promise for informing the development of responsive sexual health care and harm reduction supports for young men who use alcohol and other substances with sex.

Findings from this study identify and complicate how alcohol *specifically* features within the sexual lives of young men who use substances with sex, adding to the growing literature on sexualized substance use and chemsex/PnP. As defined in recent systematic reviews [1, 4], sexualized substance use broadly refers to sexual activities while under the influence of a wide range of substances, whereas chemsex/PnP represents a particular form of sexualized substance use that involves taking specific drugs prior to or with sex to facilitate, prolong, sustain, enhance, and/or intensify the encounter. Participants in our study tended to characterize their alcohol use in a manner that fits with the larger concept of sexualized substance use, often indicating that alcohol featured in their sexual lives in organic, unplanned ways. Still, there were instances in which alcohol was used more intentionally for chemsex/PnP, echoing other work showing that alcohol is intentionally used for the purpose of enjoying sex [13, 23]. Findings of this nature underscore opportunities to explore alcohol’s role in contexts of sexualized substance use more fulsomely, as we have argued with respect to cannabis [22], another widely used substance that is not always included under conceptual umbrellas of “sexualized substance use.” Ultimately, investigating the full spectrum of substances—inclusive of but not limited to alcohol—that young men use with sex is needed to inform the development of interventions that can more comprehensively respond to this group’s evolving contexts of sexualized substance use.

Young men in this study used alcohol to experiment with sex and sexuality, and to mitigate feelings of shame and anxiety associated with forms of non-(hetero)normative sex. These alcohol-focused findings echo insights from another recent analysis of sexual minority men’s sexualized substance use [24], wherein the authors posited that alcohol (and cannabis) are commonly used to mediate experiences of anxiety and sociability. This prior study found that young sexual minority men consume alcohol (among other substances) to ease social and sexual interactions and to reduce inhibitions, including to “provide a release from heteronormative discourses that raised [men’s] anxieties and self-doubts toward their own desires” [24]. The present analysis corroborates and extends these findings, adding—alongside other studies [13, 25]—that cisgender, straight young men also use alcohol to feel more confident and to facilitate engagement in sexual practices they might not partake in sober, including rough(er) or more aggressive sex. For sexual minority men, this “social lubrication” also aligns with the literature on minority stress theory [26, 27] and sexualized substance use [22, 28–30], which together explain how men’s desires to mediate or temporarily “let go” of minoritization-driven shame and what could be considered internalized homophobia can precipitate use of substances with sex.

The young men we interviewed used alcohol to be more adventurous and to achieve sexual(ity) freedoms, allowing them to engage in new forms of play they otherwise may not try, if sober. These dynamics of enhancement and transformation in the context of substance use with (and without) sex are well described [19, 22, 28–30]. Alongside this body of scholarship are calls for further appreciating the role of pleasure in substance use research and practice [20, 21] because pleasure and its social pragmatics can greatly influence the practices of safety, care, and risk that young men and others may take up in sex involving substances. This has important implications for service delivery, underscoring the need for sexual health and primary care providers working with young men to invite and open up meaningful conversation about how they may be using substances (including alcohol) with sex, while offering education and supports that can promote safer sex/substance use [2, 31]. It is critical that care approaches here and elsewhere (e.g., school-based sexual health education) are de-stigmatizing, sex positive, and affirming [32], particularly given issues of shame and disinhibition that subgroups of young men may face, including in contexts of transgressing conservative or restrictive social and sexual (hetero)norms.

Findings from this study offer substance-specific direction for sexual harm reduction. The young men were generally knowledgeable about how to identify and actively mitigate risks related to (sexualized) substance use, yet they also indicated that using alcohol—especially to a point of heavy intoxication—could interfere with sexual function and sexual health risk-mitigation and self-care practices. Prevention and mitigation of these sorts of consequences is best achieved through leveraging interventions that are non-stigmatizing, person-centered, and realistic, including those falling under the umbrella of harm reduction and that neither expect nor rely upon abstinence from sex and/or substance use [2, 31, 33]. Detailed at length elsewhere [34, 35], harm reduction encompasses a pragmatic response and guiding approach to care that is non-judgmental and focuses first and foremost on keeping people safe whilst preventing and mitigating harm, especially harms related to substance use and sexual health. There is ample opportunity to champion harm reduction together with syndemic or integrated approaches to sexual health and substance use care with young men [5, 31]. For example, clinicians can support young men in developing personalized plans to pre-empt against risks that may arise with sex involving alcohol and other substances, such as by talking about safer sex with one’s sexual partner(s) in advance and when not heavily intoxicated, where possible, and using alcohol only in moderation and in safe spaces and with trusted partners when sex is foreseeable. This planning could also involve using other forms of contraception, employing routine pre-exposure prophylaxis (PrEP) as an HIV prevention strategy, and/or keeping condoms on hand and in places where (drunk) sex could occur [36]. Alongside these individual efforts is demand for comprehensive, low-barrier, and accessible sexual health services for young men, inclusive of contraception, STBBI testing and treatment, and HIV pre- and post-exposure prophylaxis [32].

There are strengths and limitations to this study. Our interviews with a large and heterogeneous sample—including young men with diverse sexual identities who use substances with sex—yielded rich insights about their experiences of sexualized alcohol use. Study findings hold broad relevance yet are not representative of all young men’s experiences, especially when considering that recruitment for this study was targeted toward young men who use substances with sex. Relatedly, limitations in the specificity of our study objective and interview questions hindered our capacity to analyze how young men’s experiences with sexualized alcohol use may be shaped by aspects of social identity and location, including race/ethnicity, class, and gender modality (i.e., being transgender or cisgender). Similarly, although we offered some relevant insights, this study did not focus on delineating how young men’s experiences may vary according to the configurations of substances they use with sex (e.g., alcohol use alone versus polysubstance use). This is an area for future scholarship, as is nuanced analysis of consent dynamics in sex involving alcohol and other substances. Indeed, the complexity and scope of the issue of consent in this context warrant its own investigation, which our team is currently undertaking in a forthcoming critical discourse analysis. Finally, there is opportunity for more inclusive and/or targeted research into sexualized alcohol use with groups not represented in the present study, including women, non-binary people, and older men.

## Conclusion

This study has demonstrated that young men generally distinguish their use of alcohol with sex from sexualized substance use, per se, yet that alcohol still features in their sexual lives in complex, sometimes generative ways. Consuming alcohol with sex can offer important “social lubricant” and disinhibiting effects to young men. These effects can support experimentation with sexual(ity) freedoms but can also pose sexual risks and consequences. Together, these findings underscore that the sexualized use of substances must be understood, and responded to, in a manner that considers the full spectrum of substances—inclusive of but not limited to alcohol—and associated benefits and risks that feature in young men’s sexual lives. Harm reduction approaches are well suited to supporting young men to achieve these benefits whilst reducing potential risks, and there is a wealth of opportunities to champion harm reduction within the context of integrated substance use and sexual health care. Here, sexual health and primary care providers working with young men should invite and open up meaningful conversation about how they may be using substances (including alcohol) with sex, while offering de-stigmatizing, sex-positive, and affirming education and supports to promote safer sex and substance use.

## Data Availability

The dataset analyzed during the current study is not publicly available due to it containing information that could compromise research participant privacy and consent, but it is available from the corresponding author on reasonable request.

## Abbreviations

CDN: Canadian dollar
PrEP: Pre-exposure prophylaxis
PnP: Party and play
STBBI: Sexually transmitted and bloodborne infection
